# Association between Serum Copper Status and Working Memory in Schoolchildren

**DOI:** 10.3390/nu7095331

**Published:** 2015-08-27

**Authors:** Guoping Zhou, Xiaopeng Ji, Naixue Cui, Siyuan Cao, Chang Liu, Jianghong Liu

**Affiliations:** 1 Jintan People’s Hospital, Changzhou 213200, China; E-Mail: guopingzhoujintan@gmail.com; 2 School of Nursing, University of Pennsylvania, Philadelphia, PA 19104, USA; E-Mails: jixiaop@nursing.upenn.edu (X.J.); naixuec@nursing.upenn.edu (N.C.); caos@sas.upenn.edu (S.C.); 3 Psychology Department, Nanjing Norm University, Nanjing 210097, China; E-Mail: liuchang@njnu.edu.cn

**Keywords:** serum copper, micronutrient, working memory, cognition

## Abstract

Trace elements such as copper are essential micronutrients. Traditionally, copper has been studied in the context of micronutrient deficiencies. Recent studies in both animals and humans, however, have revealed that elevated blood copper can also have adverse effects on cognitive function since free copper can cross the blood-brain barrier and subsequently impose oxidative stress to neuronal cells. However, most of these human studies were conducted in adult populations with and without cognitive decline, and there are few studies on the effect of excess copper on cognitive function in children. This project seeks to look at the effects of elevated copper levels on cognitive development in a population of school age children (ages 10–14 years with mean age of 12.03 years and standard deviation (SD) of 0.44) from Jintan, China. Briefly, serum copper levels and working memory test scores were collected from a sample of 826 children with a mean serum copper level of 98.10 (SD 0.75). Copper level was considered as a categorical variable (taking the first group as those with as ≤84.3 μg/dL, the second group as >84.3 and ≤110.4 μg/dL, and the third group as >110.4 μg/dL with the cut-off values defined by the first and third quartiles of the sample). Results showed a significant association between high copper levels (>110.4 μg/dL) and poorer working memory in boys but this association was not seen in lower copper levels in either sex. These results suggests that in school age children, like in adults, elevated copper levels have the potential to adversely affect cognition.

## 1. Introduction

Micronutrients are known to support neurotransmitter synthesis and brain function [1]. Given rapid brain growth and development throughout childhood [2], the impact of micronutrient deficiency on brain development and neurocognitive function in child populations has been increasingly investigated. Among the micronutrients, iron deficiency has been linked to alterations in neuronal metabolism in the hippocampus and prefrontal projections where memory processing occurs [3]. The beneficial roles of zinc-supplementation on neuropsychological performance, such as reasoning and psychomotor capacity, have also been shown in Chinese and Mexican-American children [4].

While the relationship between cognitive function and micronutrient iron and zinc levels has received significant attention in the past few decades, few studies have specifically examined the relationship between copper and neurocognition in humans. Copper is involved in several enzyme systems and consequently influences immune cell function, collagen and elastin synthesis, and neurotransmitter generation [5]. Copper levels, therefore, have been hypothesized to be correlated with brain physiology [6] and neurocognitive function in humans [7,8]. The Recommended Daily Allowances (RDA) for copper for adults is 0.9 mg per day [9]. However, excessive copper levels may result in cytotoxicity [10], and cognitive problems [11]. A recent meta-analysis demonstrated that serum copper was slightly but significantly increased in patients with Alzheimer’s Disease relative to healthy controls, suggesting a possible role for copper in cognitive impairment [12]. Epidemiological studies in normal populations support this relationship by showing an association between high serum copper levels and low cognitive performance in older adults [7] and the general adult population [13]. This predictive effect of copper on cognition may interact with dietary level of saturated or trans-fats. In a large community study, accelerated cognitive decline was found in persons whose diets were high in copper and saturated and *trans* fats, but high copper intake alone did not show such trend [14].

Although studies have suggested the involvement of high serum copper levels in cognitive impairment in humans, little evidence is available regarding to the association of cooper status and cognitive function, in particular working memory, among children. Working memory (WM) represents an important cognitive function essential to childhood development and daily functioning, and therefore has important implications for learning ability and academic development in childhood. Specifically, working memory is a limited system that allows for the temporary storage and manipulation of information, and it in turn supports complex cognitive activities [15]. Poor working memory in children has been linked to inattention and short attention spans [16], impaired executive functions and classroom behavioral problems [17], as well as low academic achievement [18]. It is therefore important to understand potentially modifiable factors of poor working memory in children.

This paper aims to fill in the gap by testing the relationship between serum copper levels and working memory outcomes in normal school age children from Jintan, China. Understanding the possible role of serum copper levels in child working memory may inform future interventional studies that address early health risk factors for cognitive impairment and ultimately improve learning ability in children.

## 2. Materials and Methods

### 2.1. Participants and Procedures

The current study was part of a larger community cohort study that includes 1656 Chinese children (55.5% boys, 44.5% girls) from Jintan, China. The subjects were initially recruited in spring 2005 from four preschools in Jintan and have been followed in the past ten years. Details of this cohort study have been described in a previous publication [19]. The Jintan Cohort study is an on-going prospective longitudinal study with the aim of exploring early health risk factors in the development of child cognition and behavior. When the cohort children were about age 12 years and in their last month of sixth grade, they were invited to participate in wave II of data collection, which spanned from 2011–2013. A total of 1121 participants provided serum samples, of whom 826 completed working memory tasks. Thus, the final available data for this study consists of 826 children (53.2% boys, 46.8% girls) with a mean age of 12.02 ± 0.44 (range 10–14) years. These children also completed the Chinese version of the Wechsler Intelligence Scale for Children-Revised (WISC-R), which measures Intelligence Quotient (IQ) scores. All children in the sample had an IQ equal to or higher than 70, indicating that all the children in sample had normal cognitive states with regards to intelligence.

### 2.2. Measures

#### 2.2.1. Collection and Analyses of Blood Copper Samples

Trained pediatric nurses collected blood specimens at fast during the summer of 2011 and 2013; at the time of sample collection, the children were in their last month of sixth grade. Approximately 0.5 mL of venous blood was collected in a lead-free Ethylenediaminetetraacetic acid (EDTA) tube for copper, zinc and iron analysis. Samples were frozen and shipped to the Research Center for Environmental Medicine of Children at Shanghai Jiaotong University for the analysis. Specimens remained frozen at −20 °C until analysis. Blood concentration of copper was determined by atomic absorption spectrophotometry (BH model 5.100 manufactured by Beijing Bohu Innovative Electronic Technology Corporation), with duplicate readings taken with an integration time of 2 [20]. In addition to copper, blood levels of iron and zinc were also measured.

For the purposes of this study, quartile levels of the entire copper data set were used to define low, intermediate, and high levels of copper. Specifically, low copper level was defined as ≤first quartile (Q1 = 84.3 μg/dL), intermediate copper level was defined as >Q1 (84.3 μg/dL) and ≤third quartile (Q3 = 110.4 μg/dL), and high copper level was defined as >Q3 (110.4μg/dL).

#### 2.2.2. Working Memory Tasks

Working memory ability was assessed using four subtests of the Working Memory Measurement Software and details on the validity are given by [21,22]. Briefly, three subtests were used to measure the capacity of visuospatial working memory; these subtests were dot trajectory (the participant needed to recall a dot’s path across a grid), dot memory (the participant to reproduce a pattern of dots on a grid), and box transform memory (the participant needed to reproduce a pattern of darkened blocks on a screen) [21]. One subtest was used to measure the capacity of numeric working memory [21]; this test was digit span (the participant needed to retain and recall a string of numbers that appeared on the screen). Each of these working memory tasks required the participant to retain and recall content information displayed on a computer screen following a certain period of time after the initial display (this waiting time ranged from 3–5 s depending on the task). The final score of each task is the maximum number of the recalled dots, blocks or digits. High scores indicate better working memory capability.

The working memory test was implemented around the same time period as when the blood was drawn for copper analysis at the laboratory in Jintan People’s Hospital.

#### 2.2.3. Covariates

Children completed a questionnaire with information on sex, age, and maternal and paternal education levels. In addition to sociodemographic variables, serum iron and zinc were used as covariates due to the possible links with cognitive development [23].

### 2.3. Statistical Analysis

Sample characteristics were summarized by descriptive statistics, specifically with measures of central tendency. Since the majority of the children were age 11 (*n* = 431, 52.2% of total sample) and 12 (*n* = 368, 44.6% of total sample) years and less than 4% were either younger than 11 or older than 12 years old, we categorized children into two age groups: 10–11 years and 12–14 years. Serum copper level was categorized using Q1 (84.3 μg/dL) and Q3 (110.4 μg/dL) as cutoffs. Student’s *t* test, chi-square test and one-way analysis of variance (ANOVA) were used to examine the association between working memory and sample characteristics without controlling for other variables. Post hoc Sheffe test was used if there was significant difference among groups in ANOVA. We constructed a latent variable for working memory from the four observable variables (*i.e.*, scores on the four working memory tasks) using confirmatory factor analysis (CFA). The criteria for a good model were (1) the chi square test was not significant; (2) the comparative fit index (CFI) and nonnormed fit index (NNFI) were greater than 0.95; and (3) the root mean squared error of approximation (RMSEA) was less than 0.05. The latent variable score was calculated conditional on the four observed variables used in the model, and was used in the regression analysis to test the relationship between working memory and serum copper level; this analysis adjusted for covariates using a generalized linear model. Next, the interaction term of sex and serum copper level was added into each of the four models to test the moderating effect of children’s sex in these relationships. Generalized linear model (GLM) analyses were clustered at preschool levels when children were initially recruited to adjust the standard error. The α level was set at 0.05. Data were analyzed using Stata for Windows Version 13.0 (StataCorp LP, College Station, TX, USA).

## 3. Results

### Sample Characteristics and Working Memory

More than one third of the parents had college or graduate education. The mean levels of serum copper, iron and zinc were 98.10 ± 21.48 μg/dL, 116.90 ± 50.04 μg/dL, and 88.87 ± 16.01 μg/dL, respectively. More boys than girls had copper level higher than 110.4 μg/dL (χ^2^ = 14.15, d*f* = 1, *p* = 0.001), which is Q3 of copper levels in the entire data set. The mean scores for the four working memory test score were 8.29 ± 2.12 for dot trajectory, 6.25 ± 1.5 for digit span, 5.93 ± 1.90 for dot memory and 4.77 ± 1.18 for box transform memory. See Table 1.

**Table 1:** Sociodemographic characteristics and descriptive statistics of single working memory test score.

	*n* (%)/Mean ± Standard Deviation
Sex	
Girls	387 (46.8)
Boys	439 (53.2)
Age	
10–11 years	437 (52.9)
12–14 years	389 (47.1)
Mother’s education ^#^	
1 Middle school or less	345 (42.2)
2 High school	190 (23.2)
3 College or higher	283 (34.6)
Father’s education ^#^	
1 Middle school or less	247 (3.2)
2 High school	238 (29.1)
3 College or higher	334 (40.8)
Categorical serum copper	
1 Q1: ≤84.3 μg/dL	208 (25.6)
2 Q1–Q3: >84.3, ≤110.4 μg/dL	413 (5.8)
3 Q3: >110.4 μg/dL	191 (23.5)
Serum Iron	116.90 ± 50.04
Serum Zinc	88.87 ± 16.01
Working memory test score	
Dot trajectory	8.29 ± 2.12
Digit span	6.25 ± 1.5
Dot memory	5.93 ± 1.90
Box transform memory	4.77 ± 1.18

^#^ variables have system missing data.

**Figure 1:**
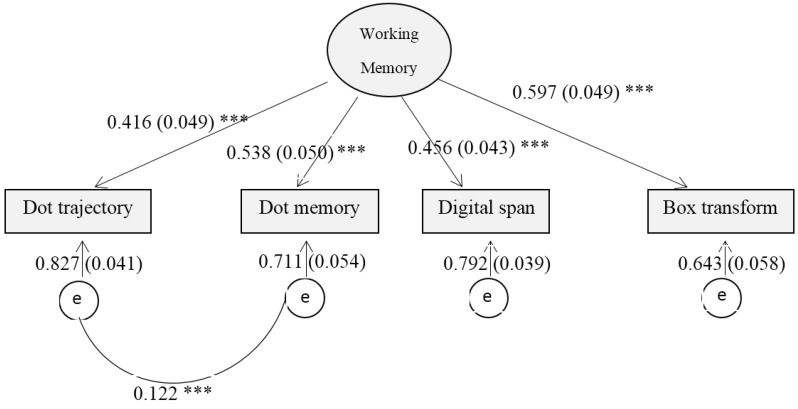
The confirmatory factor analysis of working memory using structural equation modeling. Numbers on each path from latent variable (Working Memory) to the observed variables represent standardized factor loading (standard error). Numbers on the arrows from the error terms (e) to the observed variables represent covariance of error terms. Numbers on the bottom represent the correlation between the two error terms. *** *p* < 0.001.

We conducted a confirmatory factor analysis to verify that the latent variable model was a good fit to the data (χ^2^ = 2.296, d*f* = 1, *p* = 0.130, CFI = 0.996, NFI = 0.973 and RMSEA = 0.040). The estimated parameters can be seen in Figure 1. Latent working memory scores were compared across different sociodemographic variables and summary statistics are presented in Table 2. Briefly, latent working memory score was not different between sex (*p* > 0.05) and across serum copper levels (d*f* = 2, *p* > 0.05). Children with parents that had college or higher level of education scored significantly higher on latent working memory than those whose parents had high school or lower education (d*f* = 2, *p* values < 0.001).

**Table 2:** The relationship between sociodemographic characteristics and latent working memory.

	Latent Working Memory	t/F	Kendall’s Correlation
Sex		0.2751	
Girls	0.01 ± 0.67		
Boys	0.001 ± 0.69		
Age		0.84	
10–11 years	−0.01 ± 0.69		
12–14 years	0.03 ± 0.67		
Mother’s education ^#^		2.64 *** (Post hoc Sheffe test: 3 > 1 ***, 3 > 2 **)	
1 Middle school or less	−0.13 ± 0.63		
2 High school	−0.03 ± 0.59		
3 College or higher	0.21 ± 0.75		
Father’s education ^#^		1.64 *** (Post hoc Sheffe test 3 > 1 ***, 3 > 2 *)	
1 Middle school or less	−0.11 ± 0.61		
2 High school	−0.03 ± 0.63		
3 College or higher	0.13 ± 0.75		
Categorical serum copper		1.74	
1 Q1: ≤84.3 μg/dL	0.01 ± 0.69		
2 Q1–Q3: 84.3, 110.4 μg/dL	0.05 ± 0.71		
3 Q3: >110.4 μg/dL	−0.06 ± 0.62		
Serum Iron			0.011
Serum Zinc			−0.003

* *p* < 0.05, ** *p* < 0.01, *** *p* < 0.001. ^#^ variables have system missing data.

**Figure 2:**
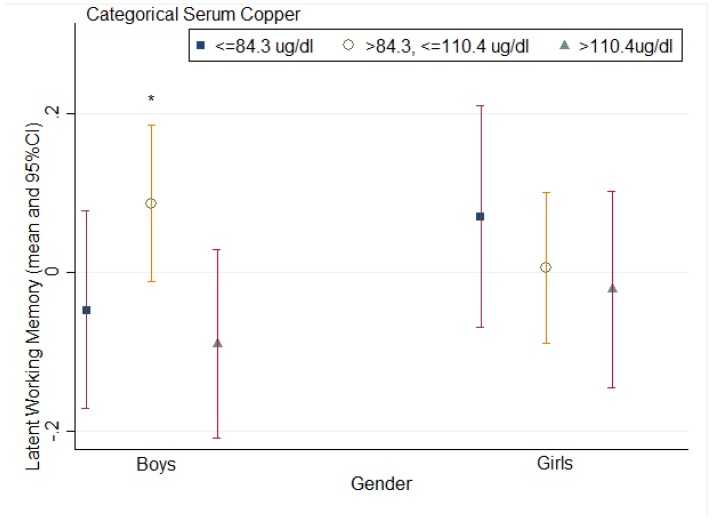
The mean and confidence interval of dot memory score by sex and serum copper level (Q1: ≤84.3 μg/dL, Q1–Q3: >84.3 μg/dL, ≤110.4 μg/dL, and Q3: >110.4μg/dL). * Significant difference between Q1–Q3: >84.3, ≤110.4 μg/dL and Q3: >110.4 μg/dL.

Table 3 depicts results from the GLM analysis. Children who had serum copper >110.4 μg/dL (Q3) consistently scored lower on latent working memory than those with serum copper levels between Q1–Q3: >83.4 μg/dL, ≤110.4 μg/dL (unstandardized regression coefficient (b) = −0.099, robust standard error (s.e.) = 0.009, *t* = −10.99, *p* = 0.008, 95% CI (−0.138, −0.060)) after controlling for sex, parental education, serum iron level and serum zinc level. After the interaction term of child’s sex and categorical serum copper was added into the model, there was a significant moderating effect of sex (F = 521.64, *p* = 0.0019) in the relationship between categorical serum copper and latent working memory. Boys in the group of categorical serum copper >Q3: 110.4 μg/dL scored significantly lower on latent working memory than those in the group of categorical serum copper between Q1–Q3: >83.4, ≤110.4 μg/dL (b = −0.143, s.e. = 0.010, *p* = 0.005). The differential effect of categorical serum copper >Q1 and categorical serum copper between Q1–Q3 on working memory was lower among girls but was not significant (b = −0.049, s.e. = 0.013, *p* = 0.066). There is a significant sex difference of this differential effect (*t* = −30.76, *p* = 0.001). See Figure 2.

**Table 3:** The regression models of association of working memory with copper.

Latent Working Memory	*b* (Robust s.e.)	*p* Values	95% CI
Categorical serum copper			
Q1: ≤84.3 μg/dL	0.097 (0.053)	0.067	(−0.007, 0.201)
Q1–Q3: >84.3, ≤110.4 μg/dL	0.099 (0.009)	<0.001	(0.082, 0.117)
Q3: >110.4 μg/dL	Ref.		
Sex			
Girls	0.013 (0.019)	0.490	(−0.024, 0.049)
Boys	Ref.		
Age			
10–11 years	−0.073 (0.032)	0.023	(−0.137, −0.010)
12–14 years	Ref.		
Mother’s education			
Middle school or less	−0.307 (0.057)	<0.001	(−0.551, −0.062)
High school	−0.208 (0.051)	<0.001	(−0.429, 0.013)
College or higher	Ref.		
Father’s education			
Middle school or less	−0.087 (0.046)	0.056	(−0.285, 0.111)
High school	−0.070 (0.046)	0.120	(−0.268, 0.126)
College or higher	Ref.		
Serum Iron	0.00003 (0.00004)	0.451	(−0.00005, 0.0001)
Serum Zinc	0.00008 (0.0002)	0.603	(−0.0002, 0.0004)

b: unstandardized regression coefficient; s.e.: standard error; CI: confidence interval; Ref.: Reference level.

## 4. Discussion

To our knowledge, this study represents one of the first examinations of the relationship between serum copper levels and working memory in children aged 10–14 years. There are several key findings in this study. Firstly, a significant association was found between high copper levels and poorer working memory in children; specifically, this association was seen in boys and not in girls. Secondly, there was no significant difference in working memory between children with medium levels of serum copper and those with low levels of serum copper.

Copper is essential to brain function, but copper can be toxic if the cellular concentration exceeds the metabolic requirement [24], which is supported by the present findings. For example, a previous study also showed an inverse relationship between plasma copper levels and cognitive function among an elderly community population in rural China [25]. Researchers have also reported the relationship between impaired cognition and relative high levels of serum copper in individuals with Alzheimer’s Disease [26].

The mechanism of cognitive impairment related to high copper levels is possibly due to copper’s toxicity to brain function. Specially, the redox-active Cu(II) facilitates *amyloid*-β peptide (Aβ)-mediated oxidative damage to brain cells and thus potentiates neurotoxicity exhibited by Aβ [27,28]. Additionally, copper may be linked to working memory through its effects on attention [13]. It has been found that copper tends to cumulate to a higher level in brain areas associated with attention, including the prefrontal cortex, nucleus caudatus, substantia nigra and locus coeruleus [29]. Attention facilitates target processing during both perceptual and post perceptual stages of processing, and functionally dissociated processes have been implicated in the maintenance of different kinds of information in working memory [30]. Therefore, higher copper levels may affect working memory capability through attention impairment.

We found a sex difference in the prevalence of higher copper level and the negative effect of high serum copper levels on working memory. More boys had copper levels higher than 110.4 μg/dL, and high serum copper level was significantly associated with poor working memory among boys but not girls. The variation in blood copper level may result from differences in food intake and environmental factors. Organ meats, shellfish and multivitamin supplements are the richest resources of copper [14]. The copper content from plant foods and tap water varies by soil and copper water pipes, respectively. However, the mechanism underlying the sex difference in serum copper levels remains unknown. With regard to the sex difference in copper-WM relationship, it has been suggested that males are more sensitive to the neurotoxic effects of copper. For example, an animal study assessing toxicity of copper nanoparticles (23.5 nm) *in vivo* revealed that nano-copper particles of the same mass might exert stronger toxicity to male than to female mice. However, a human study using a sample of older adults (60–94 years) found that higher plasma copper levels were related to lower cognitive function score in both women and men [7]. One possible reason for this inconsistency is that the Lam *et al.* study [7] examined general cognitive function, while the present study primarily focused on working memory; copper may affect different cognitive domains differently. Moreover, the neurobiological effect of copper on neurocognition may be different in children and elderly. It is possible that male children are more sensitive to the toxicity of high serum copper levels, which yield similar effects in elderly males and females. More studies are warranted to clarify the sex difference in the neurocognitive effect of copper.

We did not find a significant difference in working memory between children with medium copper levels and those with low levels, which may not be compatible with the concept of homeostasis. Theoretically, both copper deficiency and copper toxicity can lead to brain dysfunction. One possible reason for the insignificant finding could be that the lower copper level does not manifest immediate effects on working memory among children; instead, it may have long-term effects on working memory in adulthood and old age. Therefore, future research is necessary to examine the trajectory of the effect of copper on working memory across the life span.

It is worthwhile to point out that this preliminary exploratory study has three main limitations. The first is that this study measured total serum copper instead of free copper level. It has been suggested that free copper, which can cross the blood-brain barrier and reach the brain, plays a more important role in cognitive development than bound copper [13]. The finding that high total serum copper was related with low working memory may be driven by high free copper, which warrants further studies. Secondly, we did not adjust for dietary nutrients in the present study, such as saturated and *trans* fats, which may interact with copper and alter cognitive outcomes [14]. Third, this is a cross-sectional study and as a result, the relationship between serum copper and working memory cannot be interpreted as causative. Future studies are needed to investigate the long-term effect of copper on cognitive development.

Despite the limitations, our study represents an attempt to discuss high copper as a possible risk factor for diminished working memory in children, especially among boys. The current RDA for copper intake for children aged 10–13 years is 0.7 mg/day [9]. With the usage of tap water from copper-lined pipes and plant foods from copper contaminated soil, there is a potential for copper accumulation. Based on the precautionary principle that primary prevention is needed when there are threats to health until it is certain that it will not cause harm [31], it may be necessary to monitor children’s copper level, especially for boys, and maintain copper level within an appropriate range to avoid potential harm of high copper on working memory.

## 5. Conclusions

Copper has been linked to brain function and cognitive performance in humans [6,7]. However, few studies have specifically examined the association of cooper status and working memory in children. Our findings from a large school population of children aged 10–14 years suggest a potential role of elevated serum copper levels in cognitive impairment. Associations between high copper levels (>110.4 μg/dL) and poorer working memory are present in boys but not girls; and this association was not seen in lower copper levels in either sex. The potential adverse effect of high serum copper levels on working memory has significant implications for public health. It highlights the importance to monitor serum copper levels and intervene when a child has suboptimal copper status in order to address early health risk factors for cognitive impairment in school children.
